# Inhibition of corticotropin-releasing hormone receptor 1 and activation of receptor 2 protect against colonic injury and promote epithelium repair

**DOI:** 10.1038/srep46616

**Published:** 2017-05-11

**Authors:** Bo Li, Carol Lee, Tali Filler, Alison Hock, Richard You Wu, Qi Li, Shigang Chen, Yuhki Koike, Wan Ip, Lijun Chi, Elke Zani-Ruttenstock, Pekka Määttänen, Tanja Gonska, Paul Delgado-Olguin, Augusto Zani, Philip M. Sherman, Agostino Pierro

**Affiliations:** 1 Physiology and Experimental Medicine Program, The Hospital for Sick Children, Toronto, Ontario, Canada; 2 Division of General and Thoracic Surgery, The Hospital for Sick Children, Toronto, Ontario, Canada; 3 Cell Biology Program, The Hospital for Sick Children, Toronto, Ontario, Canada; 4 Division of Gastroenterology, Hepatology and Nutrition, The Hospital for Sick Children, Toronto, Ontario, Canada; 5 Biology Department, Burman University, Lacombe, Alberta, Canada; 6 Department of Paediatrics, University of Toronto, Toronto, Ontario, Canada; 7 Department of Molecular Genetics, University of Toronto, Toronto, Ontario, Canada; 8 Heart & Stroke Richard Lewar Centre of Excellence, Toronto, Ontario, Canada; 9 Developmental and Stem Cell Biology, The Hospital for Sick Children, Toronto, Ontario, Canada; 10 Department of Laboratory Medicine and Pathobiology, Faculty of Medicine, University of Toronto, Toronto, Ontario, Canada; 11 Faculty of Dentistry, University of Toronto, Toronto, Ontario, Canada; 12 Department of Surgery, University of Toronto, Toronto, Ontario, Canada

## Abstract

Maternal separation (MS) in neonates can lead to intestinal injury. MS in neonatal mice disrupts mucosal morphology, induces colonic inflammation and increases trans-cellular permeability. Several studies indicate that intestinal epithelial stem cells are capable of initiating gut repair in a variety of injury models but have not been reported in MS. The pathophysiology of MS-induced gut injury and subsequent repair remains unclear, but communication between the brain and gut contribute to MS-induced colonic injury. Corticotropin-releasing hormone (CRH) is one of the mediators involved in the brain–gut axis response to MS-induced damage. We investigated the roles of the CRH receptors, CRHR1 and CRHR2, in MS-induced intestinal injury and subsequent repair. To distinguish their specific roles in mucosal injury, we selectively blocked CRHR1 and CRHR2 with pharmacological antagonists. Our results show that in response to MS, CRHR1 mediates gut injury by promoting intestinal inflammation, increasing gut permeability, altering intestinal morphology, and modulating the intestinal microbiota. In contrast, CRHR2 activates intestinal stem cells and is important for gut repair. Thus, selectively blocking CRHR1 and promoting CRHR2 activity could prevent the development of intestinal injuries and enhance repair in the neonatal period when there is increased risk of intestinal injury such as necrotizing enterocolitis.

Neonatal maternal separation (MS) is a documented model of stress in early life^1^. This model has been used to study irritable bowel syndrome (IBS) and inflammatory bowel disease (IBD) in adulthood^2^,^3^, as well as neonatal intestinal disorders^4^,^5^,^6^. Premature infants are separated from their mothers and commonly fed while in incubators. These infants experience little physical human contact, are not breastfed, and are exposed to various stress factors such as infection, mechanical ventilation, hypothermia, and hypoxia. These stresses increase their risk of developing early intestinal disorders, such as necrotizing enterocolitis (NEC). MS during the neonatal period in a mouse model can lead to significant intestinal epithelial dysfunction. We have previously shown that MS in neonatal mice changes the intestinal mucosal morphology, increases trans-cellular permeability and causes colonic inflammation^4^,^5^,^6^. In addition, changes in the microbiome are associated with MS-induced gut injury^7^. Intestinal epithelial stem cells (IESCs) expressing leucine-rich repeat containing G-protein-coupled receptor5 (Lgr5) initiate gut repair and prevent further intestinal damage resulting from various causes^8^,^9^. However, in the MS model, the induced gut injury and subsequent repair mechanism remains to be elucidated.

The brain-gut axis is a complex network which mediates communication between the central nervous system (CNS) and the gastrointestinal tract^10^. Some of its components include sensory fibers of the spinothalamic tract, parasympathetic fibers from the vagus nerve, and the hypothalamic pituitary axis (HPA) where the CNS interfaces with the endocrine system^11^,^12^. It has been shown that the brain-gut axis influences gut function, contributing to MS-induced colonic injury^13^,^14^. Corticotropin-releasing hormone (CRH) is one of the primary brain-gut axis mediators in response to MS-induced behavioural, neuroendocrine, and autonomic changes^15^. CRH is released from the hypothalamus and stimulates adrenocorticotropic hormone secretion from the pituitary gland, which in turn leads to cortisol release from the adrenal glands^15^. In addition, CRH influences the activities of intestinal cells, such as immune cells, epithelial cells, enteric neurons, and smooth muscle cells^15^. Moussauoi *et al*. have shown that hypersensitivity to corticosteroids increases intestinal permeability in a newborn rat undergoing MS^16^. CRH-induced acetylcholine release is associated with increased macromolecular permeability of the colon in neonatal rats after MS^17^.

CRHR1 and CRHR2 are the two known G-protein coupled CRH receptors^18^. Interestingly, CRHR1 promotes intestinal inflammation and angiogenesis in an animal model of colitis, but the effects of CRHR1 are reversed by CRHR2 activation^19^. This suggests a dual role of CRH in regulating mucosal injury. Our study aims to distinguish the roles of CRHR1 and CRHR2 in MS-induced intestinal injury and repair during early postnatal life (postnatal days 5–9) in mice. This knowledge will facilitate the design of experimental treatments and prevention strategies for neonatal intestinal injury.

## Results

### MS induced colonic inflammation through CRHR1

**Figure 1:**
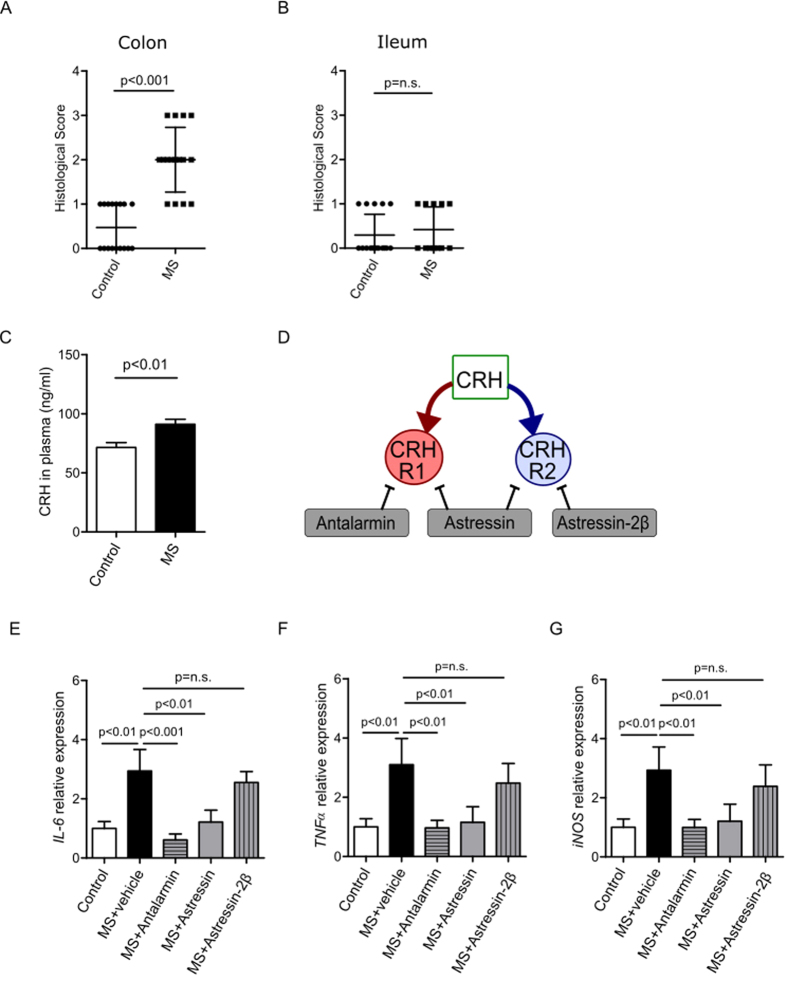
Maternal separation (MS) induced colonic inflammation through CRHR1. Histological scores of colon (**A**) and ileum (**B**) after MS were graded by three blinded investigators. MS induced histological injury in colon but not in ileum. Corticotropin-releasing hormone (CRH) levels in plasma (ng/ml) after MS were elevated compared to control (**C**). Schematic representation of CRH receptors and receptor inhibitors (**D**): Antalarmin (CRHR1 antagonist), Astressin (non-specific CRHR antagonist), and Astressin-2β (CRHR2 antagonist). The relative expressions of pro-inflammatory cytokines *IL-6* (**E**), *TNFα* (**F**) and *iNOS* (**G**) were quantified by qPCR. MS increased *IL6, TNFa* and *iNOS* levels. These effects were inhibited by Antalarmin and Astressin. Conversely, Astressin-2β did not have an effect on MS-induced inflammation. Results are presented as means, ±SD. p < 0.05 was considered significant.

Similar to what we have previously demonstrated^6^, MS induced morphological damage in the colon, but not in the ileum (Fig. 1A,B). In addition, MS induced a significant increase in CRH levels in plasma compared to controls (Fig. 1C).

To elucidate the roles of CRHR1 and CRHR2 in MS-induced gut injury, we administered: i) Astressin, a non-specific CRHR antagonist of both CRHR1 and CRHR2, ii) Antalarmin, a CRHR1 antagonist, or iii) Astressin-2β, a CRHR2 antagonist prior to MS on each day from postnatal day 5 to 9 (Fig. 1D).

Our group has shown that MS induces the expression of pro-inflammatory cytokines *IL-6, TNFα* and *iNOS* in colonic epithelium^5^. In addition, these cytokines are upregulated in neonatal intestinal diseases such as NEC^20^,^21^. In the present study, the increases in *IL6, TNFa* and *iNOS* were inhibited by pre-treatment with Astressin (Fig. 1E–G). Similarly, Antalarmin, but not Astressin-2β, prevented the MS-induced elevation in pro-inflammatory cytokines (Fig. 1E–G). These results confirm that MS induces an increase in pro-inflammatory cytokines via CRH, which can be inhibited by blocking CRHR1.

### MS-induced mucosal injury is dependent on CRHR1

**Figure 2:**
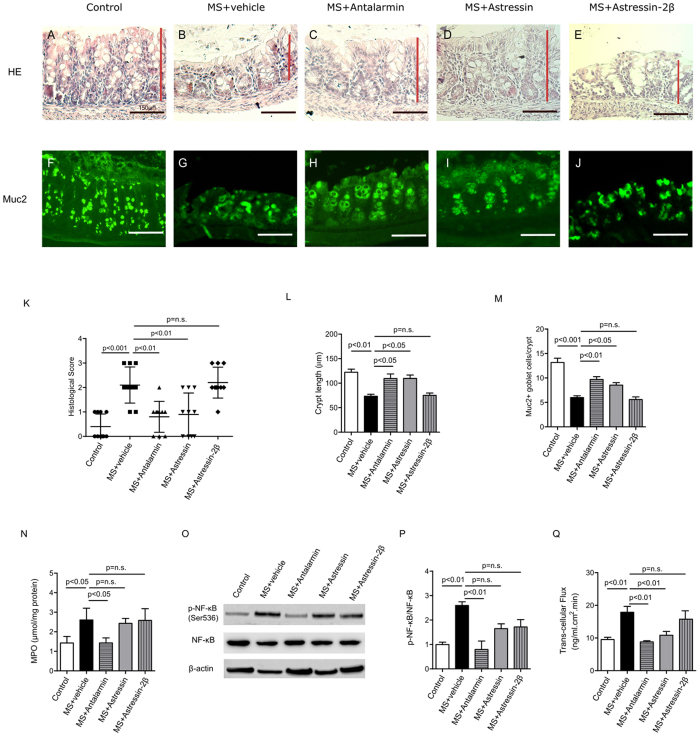
MS-induced intestinal epithelium injury was CRHR1 dependent. Photomicrographs of hematoxylin and eosin (H&E) stained (**A**–**E**) and immunofluorescence of Mucin 2 (Muc2; mucous-forming protein) (**F**–**J**) in proximal colon in all experimental groups. Histological scores (**K**) were highest in MS, demonstrated injury in MS compared to control. Treatment with Antalarmin and Astressin prevented this MS-induced colonic injury, but not by Astressin-2β. Crypt length in μm (**L**) (red lines in photomicrographs **A**–**E**) and the number of Muc2+ goblet cells per crypt (**M**) were reduced by MS compared to control, and restored to control levels following Antalarmin and Astressin treatment. Astressin-2β did not prevent these MS-induced effects. Myeloperoxidase (MPO; μmol/mg protein) expression was increased in MS group and was reduced to a level similar to control by treatment with Antalarmin but not by treatment with Astressin or Astressin-2β (**N**). Western blot analysis of NF-κB showed an increase in the phosphorylated expression of NF-κB in MS, which was prevented by Antalarmin administration, but not by Astressin or Astressin-2β (**O**,**P**). Trans-cellular flux of HRP (ng/ml.cm2.min; **Q**) measured by Ussing Chamber was increased in MS and MS + Astressin-2β groups, compared to control, but not in MS + Antalarmin and MS + Astressin groups (**P**). Results are means, ±SD. p < 0.05 was considered significant.

We further investigated the effects of CRHRs on mucosal morphology, immune activation and colonic permeability. MS caused colonic morphological damage (Fig. 2A,B,K), a reduction in crypt length (Fig. 2L), and a loss of goblet cells (Fig. 2F,G,M). However, the administration of Antalarmin and Astressin during MS improved colonic morphology (Fig. 2C,D,K), increased crypt length (Fig. 2L) and the number of goblet cells per crypt (Fig. 2H,J,M). Treatment with Astressin-2β did not rescue the MS-induced colonic injury (Fig. 2E,J).

We have reported that levels of myeloperoxidase (MPO), a pro-inflammatory enzyme highly expressed in neutrophils, are increased following MS^22^. The current results are in accordance with the above findings (Fig. 2N). In addition, administration of Antalarmin reduced MPO expression to a level similar to control. On the contrary, the other two CRH receptor antagonists, Astressin and Astressin-2β, did not exert any effects on MPO levels which remained similar to MS alone.

NF-κB plays a key role in initiating and regulating inflammation, and has been implicated in inflammatory bowel disease^23^,^24^,^25^. To elucidate whether NF-κB acts as the mediator for intestinal inflammation downstream of CRHR1, we examined its protein expression. The ratio of phosphorylated to total NF-κB was higher in the MS group compared to controls, and was rescued by Antalarmin, but not Astressin or Astressin-2β (Fig. 2O,P).

We have previously established an experimental protocol to study intestinal permeability in MS using Ussing Chamber. Only colonic trans-cellular permeability increased in the MS group^6^ and the increase was inhibited by the administration of Antalarmin and Astressin, whereas Astressin-2β did not (Fig. 2Q).

These results indicate that the CRHR1 pathway is involved in MS-associated changes in mucosal morphology, NF-κB phosphorylation, inflammation, and permeability. In contrast, the antagonist of CRHR2, Astressin-2β, did not exert any effect on morphology, immune activation and colonic permeability. Taken together, these results demonstrated that selective inhibition of CRHR1 activity with Antalarmin reduces MS-induced colonic injury.

### MS induced the activation of Lgr5+ IESCs and promoted epithelial cell proliferation

**Figure 3:**
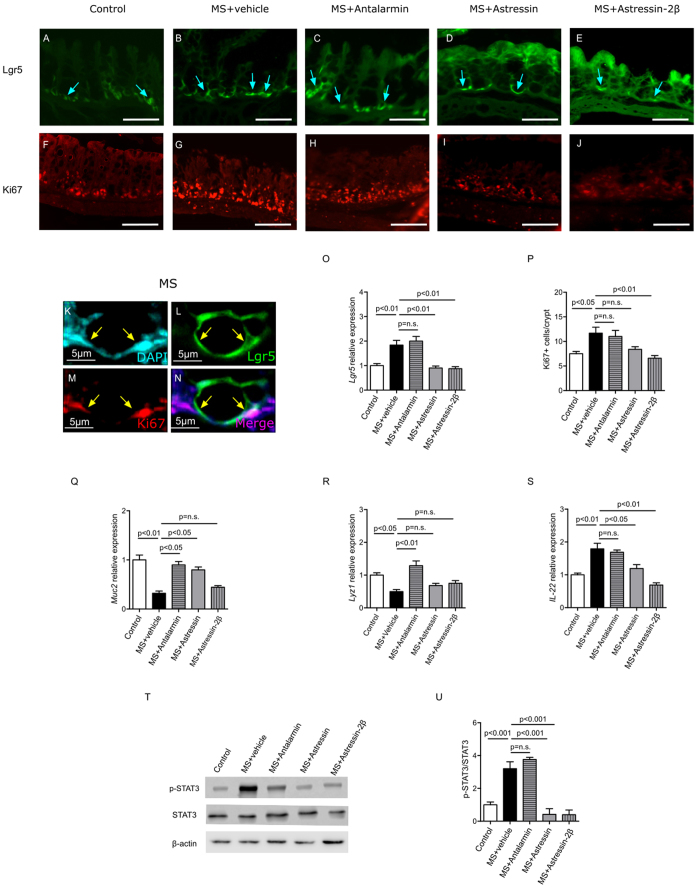
CRHR2 modulates the MS-induced increase in Lgr5+ intestinal epithelial stem cells (IESCs). Fluorescent micrographs of intestinal stem cell marker Lgr5 (**A**–**E**, blue arrows) and cell proliferation marker Ki67 (**F**–**J**) in colonic tissues of the experimental groups. Lgr5 positive cells expressing Ki67 are shown in higher magnification (**K**–**N**, yellow arrows). Co-localization of Lgr5 and Ki67 markers in the intestine of pups subjected to MS suggested that Lgr5+ intestinal stem cells are proliferative. Relative gene expressions of *Lgr5* (**O**) and the number of Ki67+ proliferating cells per crypt (P) in the colon were significantly increased by MS compared to control, and this increase was observed when Antalarmin was administered. In contrast, this increase was prevented by pre-treatment with both Astressin and Astressin-2β. Relative gene expressions of epithelial differentiation marker *Muc2* (**Q**) and *Lyz1* (**R**) are shown, The MS-induced decrease in *Muc2* and *Lyz1* expression was rescued by Antalarmin and Astressin, but not Astressin-2β. Relative gene expression *IL-22* (**S**) and western blot of phosphorylated STAT3 (**U**) are shown. *IL-22* and phosphorylated STAT3 increased in the MS group compared to the control; however, these elevations were both inhibited by Astressin and Astressin-2β, indicating the important role of CRHR2 in tissue repair in response to MS-induced injury. Results are means, ±SD. p < 0.05 was considered significant.

We hypothesized that in response to MS, intestinal stem cells induce gut repair and prevent further intestinal damage. To test this hypothesis, we performed MS experiments in Lgr5-EGFP-IRES-creERT2 pups, as their Lgr5+ intestinal epithelial stem cells (IESCs) are GFP-positive and can be easily visualized. After MS, the expression of Lgr5+ IESCs was increased at the base of the crypts, compared to controls (Fig. 3A,B,O, blue arrow). Moreover, Ki67+ proliferating cells were also increased in the MS group (Fig. 3F,G,P). Double staining of Lgr5 and Ki67 in the intestine of pups subjected to MS indicated that Lgr5+ IESCs were proliferative (Fig. 3K–N, yellow arrow). These results indicated that Lgr5+ IESCs were activated and proliferated during MS, representing a compensatory mechanism in response to MS-induced colonic injury.

### CRHR2 mediated MS-induced increases in Lgr5+ IESCs and intestinal repair through IL-22

To distinguish the roles of CRHR1 from CRHR2 in MS-induced activation of Lgr5+ IESCs, we compared the effects of specific CRHR inhibitors. Lgr5+ IESCs and *Lgr5* mRNA levels in the colon were significantly increased by MS compared to control, and this increase was also observed when Antalarmin was administered (Fig. 3C,O). In contrast, the increase in Lgr5+ IESCs and *Lgr5* mRNA levels was inhibited by Astressin and Astressin-2β (Fig. 3D,E,O). Similarly to Lgr5, the number of Ki67+ proliferating cells increased in Antalarmin, but not in Astressin-2β (Fig. 3H,I,J,P).

Thus, selective inhibition of CRHR2 blunts Lgr5 activation during MS, indicating a role for CRHR2 signaling in intestinal repair. The selective inhibition of CRHR1 did not alter *Lgr5* expression. This would potentially allow specific targeting of CRHR1 to down regulate colonic injury, while maintaining the CRHR2-mediated repair mechanism.

To further assess the epithelial differentiation potential of Lgr5+ IESCs in response to MS, we quantified the expression of differentiation marker genes of goblet cells, the predominant type of cell in the colon, and Paneth cells. The mRNA levels of the goblet cell marker *Muc2* (Fig. 3Q) and the Paneth cell marker *Lyz1* (Lysozyme1, Fig. 3R) were significantly lower in the MS group, compared to controls, but were significantly increased in mice treated with Antalarmin. In contrast, in the Astressin-2β treated group, *Muc2* and *Lyz1* levels did not fully recover to control levels (Fig. 3Q,R).

To assess the gut repair mechanisms, we measured levels of *IL-22* mRNA and the ratio of phosphorylated STAT3 to total STAT3, which are factors implicated in intestinal epithelial tissue regeneration and remodeling^26^,^27^. *IL-22* (Fig. 3S) and phosphorylated STAT3 (Fig. 3T,U) increased in the MS group compared to the control group. The CRHR2 blocker Astressin-2β inhibited such increase.

Thus, CRHR2 is required for stem cell activation, and epithelium proliferation and differentiation during MS, and mediates intestinal repair in response to MS-induced injury.

### CRHR1 restored MS-induced microbiome changes

**Figure 4:**
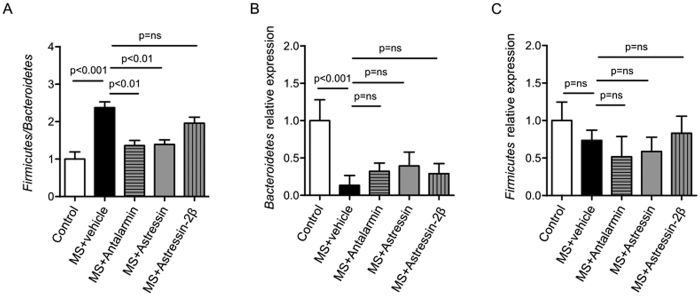
CRHR1 restored MS-induced microbiome changes. Quantitative PCR analysis of intestinal microflora composition in each experimental group. *Firmicutes to Bacteroidetes* ratio (**A**), relative expressions of *Bacteroidetes* (**B**), and *Firmicutes* (C) 16 S ribosomal RNA genes. The *Firmicutes to Bacteroidetes* ratio (**A**) was elevated by MS, and restored to control levels by Antalarmin and Astressin administration, but not Astressin-2β. The relative expression of *Bacteroidetes* (**B**) was significantly reduced following MS compared to control, and remained at this reduced level in all three treatment groups. There was no difference in *Firmicutes* levels (**C**) observed in all experimental groups. Results are expressed as means, ±SD. p < 0.05 was considered significant.

Changes in the intestinal microbiota are associated with the development of impaired gut function during MS^7^. To investigate the role of CRHRs in alterations of the microbiome during MS, we compared the relative levels of specific microbial phyla in colonic epithelium. The ratio of *Firmicutes* to *Bacteroidetes* was significantly increased by MS, but was almost completely restored by administration of either Antalarmin or Astressin, but not Astressin-2β (Fig. 4A). The MS-induced increase in the *Firmicutes* to *Bacteroidetes* ratio was due primarily to a relative decrease in the *Bacteroidetes* phylum rather than changes in *Firmicutes* (Fig. 4B,C). These results demonstrate that MS induces changes in the colonic microbiota, and that CRHRs are involved in these alterations, particularly CRHR1.

## Discussion

**Figure 5:**
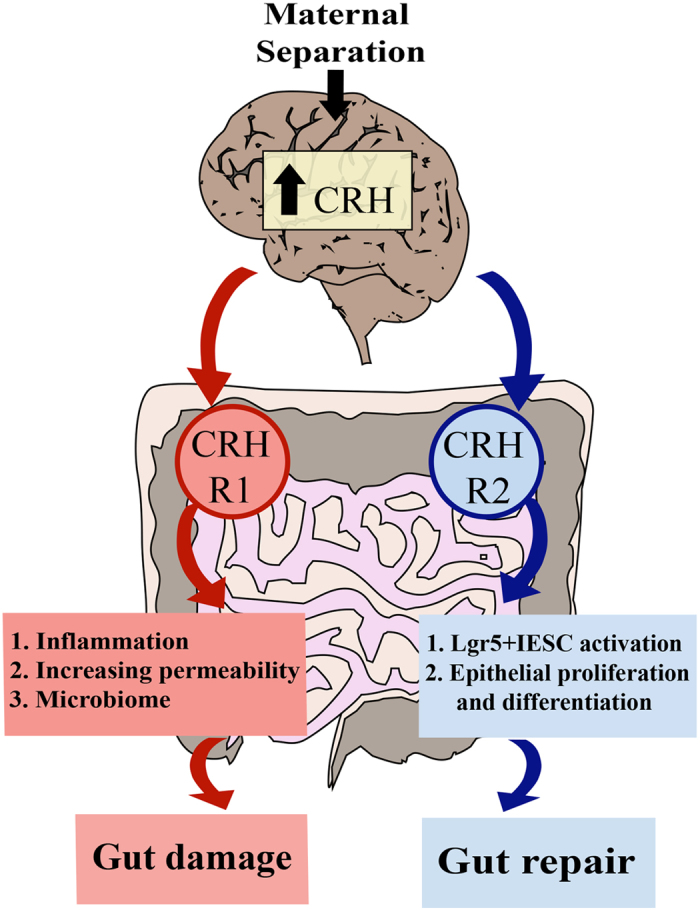
Schematic diagram of CRH in MS-induced intestinal injury. Schematic diagram illustrating the role of CRH in the brain-gut axis, which is critical for the induction of colonic injury caused by maternal separation. We suggest that maternal separation is associated with an increase in CRH secretion by the hypothalamus. CRH binds to CRHR1 and CRHR2, which have opposing effects. CRHR1 is involved in the initiation of gut damage, through increased inflammation, permeability and alterations of the microbiome. CRHR2 is involved in intestinal injury repair by activating Lgr5+ IESCs and promoting epithelial cell proliferation and differentiation.

We have demonstrated that treatment with the CRHR1 antagonist Antalarmin protects against colonic injury following MS by preventing changes in colonic morphology, inflammation, permeability and microbiota. Astressin-2β, a CRHR2 antagonist, inhibited intestinal stem cell activity and subsequent epithelial cell repair induced by MS. These results indicate that the interactions between the brain and the gut are modulated by opposing effects of CRHR1 and CRHR2. CRHR1 induces colonic injury while CRHR2 promotes healing and repair of the intestine (Fig. 5).

Stress during early life plays an important role in gut disorders such as NEC^28^; however, there is limited information on the role of CRHRs in mediating neonatal intestinal injury or intestinal repair. Our data suggests the opposing roles for CRHR1 and CRHR2, in agreement with previous experiments in adult mice with gut injury such as colitis^19^,^29^,^30^. Inhibition of CRHR1 has been shown to dramatically reduce intestinal injury in animal models of colitis^19^,^29^,^30^. Herein, we demonstrated that blocking CRHR1 prior to intestinal injury protects mouse pups from MS-induced damage, changes in gut morphology, mucosal inflammation, and barrier function. We found that the elevation in MPO expression induced by MS was rescued by the CRHR1 antagonist but not by the CRHR2 antagonist. Additionally, our MS gut injury model revealed a role of CRHR2 in mucosal repair. Activation of CRHR2 inhibits CRHR1-induced intestinal inflammation, as well as endogenous and inflammatory angiogenesis^19^. Furthermore, CRHR2 antagonists aggravate disease, delay healing, and decrease epithelial cell proliferation after DSS-induced colitis^26^.

It has been shown that Lgr5+ IESCs are responsible for epithelial cell renewal and gut repair by giving rise to functional Paneth cells and goblet cells^9^. MS markedly alters the distribution of intestinal epithelial secretory cells in the duodenum in association with changes in IESCs^31^. We have demonstrated that in MS, Lgr5+ IESCs are activated at the base of crypts where there is an increase in epithelial cell proliferation. CRHR2 is involved in epithelial tissue repair by promoting Lgr5+ IESCs. CRHR2 activation can initiate intestinal wound repair via phosphorylation of STAT3^26^, which subsequently increases the expression of *IL-22*^27^. A recent study suggested that *IL-22* directly regulates IESCs during epithelial regeneration after tissue damage^32^. We confirmed that MS, mediated by CRHR2, increases phosphorylation of STAT3 and *IL-22*, resulting in increased expression of Lgr5+ IESCs. Components of this pathway can be targeted clinically for treatment of injuries associated with Lgr5+ IESC dysfunction, such as NEC in stressed prematurely born infants.

Mice in the neonatal period are highly prone to alterations in their microbiota composition, resulting from exposure to changes in the environment^33^. These early microbial changes can be durable and persist into adulthood^7^. For these reasons, we investigated changes in the microbiota following MS in early life, and found that MS changes the microbiota by decreasing levels of *Bacteroidetes*, resulting in an increased *Firmicutes* to *Bacteroidetes* ratio. This finding is in line with studies of IBS in humans, in which *Bacteroidetes* is reduced, along with microbial diversity^34^,^35^. The microbiota of IBS patients compared to controls has a two-fold increase in the ratio of *Firmicutes* to *Bacteroidetes*^36^,^37^. In addition, IBS patients have increased expression of TLR4 and TLR5, which initiate innate immune responses through microbial stimuli^38^. Probiotics have been shown to stabilize intestinal microbiota in clinical trials^39^, alleviate inflammation^40^, and improve IBS symptoms^41^. We found that the CRHR1 selective antagonist Antalarmin and the non-selective CRHR antagonist Astressin can both partially rescue microbial dysbiosis caused by MS, while the CRHR2-selective antagonist Astressin-2β cannot. These results emphasize the potential therapeutic effects of Antalarmin in maintaining microbiota balance during stress induced by MS. However, at this point, we cannot rule out the possibility that disturbances in microbiota diversity altered CRH signaling. A full characterization of the role of microbiota and CRH signaling in neonatal intestinal disorders is needed.

Blocking CRHR1 directly improves motility, endocrine secretion, immune response, and inflammation in the mouse gut^19^,^29^,^30^,^40^,^41^. Our study demonstrated that the CRHR1 antagonist Antalarmin reduces colonic damage by modulating the mucosal immune system, improving intestinal barrier function and stabilizing gut microbiota in a model of MS. Blocking CRHR1 with Antalarmin did not interfere with Lgr5+ IESC-mediated initiation of epithelial repair, while the CRHR2-selective antagonist Astressin-2β did. Since 2004, various CRHR1 antagonists have entered clinical trials for the treatment of depression, IBS, and social anxiety disorder. However, none of the investigated CRHR1 antagonists have successfully completed a Phase III clinical trial^42^. This discrepancy is likely related to the differences in route of administration, dose, and side effects of CRHR1 antagonists between animals and humans. Nevertheless, our study demonstrates the benefits of blocking the CRHR1 pathway in an experimental model of neonatal bowel injury.

Preterm babies fed while in incubators have been shown to experience maternal separation. Preterm birth and MS cause a myriad of complications including alterations in the gut microbial composition and changes in barrier function^43^. Although our MS model induces a localized moderate colonic injury, which is not as severe as that found in NEC, there are studies that demonstrate that MS in mouse pups causes activation of the hypothalamic–pituitary–adrenal axis (an indicator of stress), colonic epithelial barrier dysfunction and bowel inflammation^44^. This could be likened to that seen in the early stages of NEC^45^. Hence, the mechanisms by which Antalarmin affects the CRHR1 pathway could represent a novel therapeutic strategy in premature babies to prevent the progression from early/suspected to advanced NEC. It is notable that some of the CRHR1-associated beneficial effects could be specific to NEC, since the mechanism of NEC development does not resemble that of intestinal disorders that develop in adulthood. For example, MPO elevation was only found in the intestinal mucosa of NEC^46^ and IBD^47^ but not in IBS^47^ patients. Therefore, further experimental studies are needed to investigate the specific role of Antalarmin in NEC initiation and progression.

In conclusion, our study demonstrates that the brain-gut axis acts through CRH to modulate colonic injury during maternal separation. CRHR1 signaling mediates intestinal injury by promoting intestinal inflammation, affecting epithelial cell permeability and morphology, and altering the colonic microbiome in response to MS. In contrast, CRHR2 mediates the activation of intestinal stem cells and is important for epithelial repair after injury. The findings of this study suggest, therefore, that inhibiting the action of CRHR1, while promoting the activity of CRHR2 could be a novel strategy to prevent gut injury in preterm infants.

## Methods

### Animals and maternal separation

All animal experiments (no. 32238) were approved by, and followed according to guidelines and regulations from the Animal Care Committee at The Hospital for Sick Children.

Postnatal day 5 C57BL/6 mouse pups were randomly assigned to control (n = 10) and maternal separation (n = 40) groups. To establish our MS model, C57BL/6 pups were separated from their mothers at the same time each day for 3 hours between postnatal day five (P5) and day nine (P9), as described previously^1^. Control neonatal mice remained with their mothers for the duration of the study. Several litters were used and pups from each litter were randomly assigned to each of the experimental groups to eliminate potential differences between litters and litter effects. Lgr5-EGFP-IRES-creERT2 mice were obtained from The Jackson Laboratory (Sacramento, USA), to study the state of intestinal stem cells in their resulting pups after exposure to our MS model.

Pups were injected with the following drugs at the same time each day between P5 and P9, before MS occurred: (i) Antalarmin, a CRHR1 antagonist (20 mg/kg/day, subcutaneously, n = 10), (ii) Astressin-2β, a CRHR2 antagonist (150 μg/kg/day, subcutaneously, n = 10), or (iii) Astressin, a non-specific CRHR antagonist inhibiting both CRHR1 and CRHR2 (60 μg/kg/day, intraperitoneally, n = 10). Injection was performed without any complications. Mice in the MS group (n = 10) received an injection of saline placebo vehicle. Routes of administration and doses were selected according to a previous report^31^. MS, as described above, was performed immediately after injections. On P9, mice pups were sacrificed and colonic tissue was harvested.

### Intestinal morphology

Colonic tissues were fixed in 4% paraformaldehyde, embedded in paraffin, cross-sectioned (5 μm) and stained with standard hematoxylin and eosin (H&E). Goblet cells were visualized by immunofluorescence staining according to the protocol described below. Histological scores, crypt length and the number of goblet cells per crypt (average of goblet cells counted on 5 crypts) were determined by three blinded independent investigators. Injury grades were assigned based on a previous publication^6^.

### CRH measurements

Blood was collected from neck arteries and veins upon sacrifice on postnatal day 9 by decapitation and centrifuged (10 minutes, 2500xg) to harvest plasma. CRH levels were measured by enzyme-linked immunosorbent assay (ELISA) (Kamiya Biomedical, Seattle, WA). Absorbance was determined using a micro-plate reader with an optical density of 412 nm (Molecular Devices SpectraMax Gemini EM) and concentration was determined using a standard curve.

### Gene expression analysis

RNA was isolated from the proximal colon with TRIzol (Invitrogen, Carlsbad, CA). Total RNA (1 μg) was reverse transcribed by qScript cDNA supermix (Quanta Biosciences, Gaithersburg) and SYBR green-based qRT–PCR was performed with advanced qPCR Supermix (Wisent Inc., Quebec, Canada). The qPCR primer sequences (Supplementary Table S1) were taken from previous publications^22^,^48^,^49^, demonstrating primer efficiency, and were compared to the Nucleotide BLAST database for confirmation. *GAPDH* levels were similar in all experimental groups and served as control for normalization. Gene expression levels were quantified using Bio-Rad CFX Manager Software (Hercules, USA).

### Intestinal permeability

A Ussing chamber was used to measure trans-epithelial resistance and trans-cellular permeability as previously reported^6^,^50^,^51^. Briefly, fresh colon tissue specimen including the epithelium and muscle layers (no stripping was performed) were cut longitudinally to expose the lumen, and mounted into Ussing chamber (Physiologic Instruments, San Diego, CA). The trans-epithelial resistance (Rte) of the colon was measured. Unaltered Rte at the beginning and the end of the experiment indicated that the tissues remained intact during the experiment. Trans-cellular permeability to large molecules was measured by assessing the mucosal-to-serosal passage of Horseradish Peroxidase (HRP, 44 kDa) (Sigma, St. Louis, MO). 0.4 mg/ml HRP was added to the apical side and its translocation was measured on the basolateral side after 20 minutes^6^. The basolateral concentration of HRP was determined using a kinetic enzymatic assay and measuring the optical density (485 nm) with a micro-plate reader (Molecular Devices SpectraMax Gemini EM). Permeability of HRP was expressed as concentration/area/minute.

### Myeloperoxidase (MPO) assay

Protein was isolated from colonic tissue by homogenization in tissue extraction buffer (Invitrogen, CA, USA) containing Protease Inhibitor Single-Use Cocktail (Sigma). Protein concentration was determined using bicinchoninic acid protein assay (Thermo Scientific, IL, USA).

MPO is a pro-inflammatory enzyme that is highly expressed in neutrophils^22^. MPO concentrations were determined using the Colorimetric Activity Assay Kit (Sigma). Absorbance readings at 412 nm were compared to a standard curve and concentration was expressed as μmol/mg protein.

### Protein quantification

Proteins were isolated as described above and were separated by NuPAGE 4–12% BisTris gel electrophoresis and transferred to a polyvinylidene fluoride (PVDF) membrane using iBlot Gel Transfer Device (Life Technologies, MD, USA). The membrane was probed with antibodies for phosphorylated NF-κB (3031, Cell Signaling Technology CST, Danvers, MA), NF-κB (8242, CST), phosphorylated STAT3 (9145, CST) and STAT3 (4904, CST). β-actin (4967, CST) served as a loading control. Blots were developed using an ECL Plus kit (Invitrogen, CA, USA). Band intensities were quantified using an Odyssey scanner (LI-COR Biosciences, Lincoln, NE).

### Immunofluorescence

Staining for Muc2 (NB120-11197, Novus Biologicals, CO) and Ki67 (ab15580, Abcam Inc., Cambridge, MA) was performed to measure epithelial goblet cell numbers and enterocyte proliferation, respectively. Tissue sections were incubated with primary antibodies (1:500 dilution) overnight at 4 °C and with fluorescent secondary antibodies (1:1000) (Life Technologies, MD, USA) for two hours at room temperature. Positively stained cells were counted in five contiguous crypt units (for each section), and expressed as the number of positive cells per crypt.

### Microbiome

To avoid contamination, mouse pups were sacrificed in a biosafety cabinet and one fragment of the whole proximal colon and its contents (1 cm) were harvested. DNA was extracted with TRIzol (Invitrogen, Carlsbad, CA) according to the manufacturer’s instructions. DNA was reconstituted to 40ng and examined by qPCR, using qPCR Supermix (Wisent Inc., Quebec, Canada) as described above. Primers sequences for 16 S ribosomal RNA genes for *Bacteroidetes*, Firmicutes, and *Eubacteria (Universal*) obtained from a previous publication^49^, are shown in the Supplementary Table S1.

### Statistics

Results are presented as mean ± SD, as data were normally distributed (Kolmogorov-Smirnov test). Groups were compared using Student’s t-test or one-way ANOVA with Bonferroni correction as appropriate. P < 0.05 was considered significant.

## Additional Information

**How to cite this article**: Li, B. *et al*. Inhibition of corticotropin-releasing hormone receptor 1 and activation of receptor 2 protect against colonic injury and promote epithelium repair. *Sci. Rep.* **7**, 46616; doi: 10.1038/srep46616 (2017).

**Publisher's note:** Springer Nature remains neutral with regard to jurisdictional claims in published maps and institutional affiliations.

## Supplementary Material

Supplementary Information
