# Supplementary material for: Inhibition of corticotropin-releasing hormone receptor 1 and activation of receptor 2 protect against colonic injury and promote epithelium repair
Source: Sci Rep. 2017 May 11;7:46616. doi: 10.1038/srep46616 (PMC5425914; doi:10.1038/srep46616)
Supplement: Supplementary Information [file srep46616-s1.pdf]

Inhibition of corticotropin-releasing hormone receptor 1 and activation of receptor 2 protect against colonic injury and promote epithelium repair

Bo Li[1,2], Carol Lee[1,2], Tali Filler[1,2], Alison Hock[1,2], Richard You Wu[3,4], Qi Li[1,2], Shigang Chen[1,2], Yuhki Koike[1,2], Wan Ip[1,4], Lijun Chi[1], Elke Zani-Ruttenstock[1,2], Pekka Määttänen[5], Tanja Gonska[1,4,6], Paul Delgado-Olguin[1,7,8], Augusto Zani[2,9], Philip M. Sherman[3,4,10,11], Agostino Pierro[1,2,12]*

[1] Physiology and Experimental Medicine Program, The Hospital for Sick Children, Toronto, Ontario, Canada;

[2] Division of General and Thoracic Surgery, The Hospital for Sick Children, Toronto, Ontario, Canada;

[3] Cell Biology Program, The Hospital for Sick Children, Toronto, Ontario, Canada;

[4] Division of Gastroenterology, Hepatology and Nutrition, The Hospital for Sick Children, Toronto, Ontario, Canada;

[5] Biology Department, Burman University, Lacombe, Alberta, Canada;

[6] Department of Paediatrics, University of Toronto, Toronto, Ontario, Canada;

[7] Department of Molecular Genetics, University of Toronto, Toronto, Ontario, Canada;

[8] Heart & Stroke Richard Lewar Centre of Excellence, Toronto, Ontario, Canada;

[9] Developmental and Stem Cell Biology, The Hospital for Sick Children, Toronto, Ontario, Canada;

[10] Department of Laboratory Medicine and Pathobiology, Faculty of Medicine, University of Toronto, Toronto, Ontario, Canada;

[11] Faculty of Dentistry, University of Toronto, Toronto, Ontario, Canada;

[12] Department of Surgery, University of Toronto, Toronto, Ontario, Canada

Supplementary Table S1: Sequences of primer pairs used for qPCR.

Name	Forward Sequence	Reverse Sequence
IL-6 [1]	CCAATTTCCAATGCTCTCCT	ACCACAGTGAGGAATGTCCA
TNFα [1]	TTCCGAATTCACTGGAGCCTCGAA	TGCACCTCAGGGAAGAATCTGGAA
iNOS [1]	CTGCTGGTGGTGACAAGCACATTT	ATGTCATGAGCAAAGGCGCAGAAC
GAPDH [1]	TGAAGCAGGCATCTGAGGG	CGAAGGTGGAAGAGTGGGAG
Lgr5 [2]	TGCCATCTGCTTACCAGTGTTGT	ATTCCGTCTTCCCACCACGC
Lyz1 [2]	GAGACCGAAGCACCGACTATG;	CGGTTTTGACATTGTGTTCGC
Muc2 [2]	GAACGGGGCCATGGTCAGCA	CATAATTGGTCTGCATGCC
IL-22 [3]	CCCAGTCAGACAGGTTCCA	TGATCTCTCCACTCTCTCCA
Bacteroidetes [3]	CRAACAGGATTAGATACCCT	GGTAAGGTTCCTCGCGTAT
Firmicutes [3]	TGAAACTYAAAGGAATTGACG	ACCATGCACCACCTGTC
Universal [3]	AAACTCAAAKGAATTGACGG	CTCACRRCACGAGCTGAC

1 Li, B. *et al.* Intestinal epithelial injury induced by maternal separation is protected by hydrogen sulfide. *Journal of pediatric surgery* **52**, 40-44 (2017).

2 de Groot, R. E. *et al.* Retromer dependent recycling of the Wnt secretion factor Wls is dispensable for stem cell maintenance in the mammalian intestinal epithelium. *PloS one* **8**, e76971 (2013).

3 Grasberger, H. *et al.* Increased Expression of DUOX2 Is an Epithelial Response to Mucosal Dysbiosis Required for Immune Homeostasis in Mouse Intestine. *Gastroenterology* **149**, 1849-1859 (2015).
